# Assessment of the effects of rehabilitation of hand function using a biometrics device in people after stroke – a randomized controlled trial

**DOI:** 10.3389/fneur.2025.1643336

**Published:** 2025-09-16

**Authors:** Justyna Leszczak, Bogumiła Pniak, Grzegorz Gazda, Agnieszka Guzik

**Affiliations:** ^1^Faculty of Health Sciences and Psychology, Collegium Medicum, Institute of Physiotherapy, University of Rzeszów, Rzeszów, Poland; ^2^Excelsior Health and Rehabilitation Hospital, Iwonicz-Zdrój, Poland

**Keywords:** rehabilitation, upper limb, stroke, biometrics, hand function

## Abstract

**Purpose:**

The aim of the study was to assess the effects of rehabilitation using the Biometrics device on the re-education of hand function in late stroke patients.

**Methods:**

The data were collected from 1 August 2022 to 28 February 2023. The study was conducted among 120 people after stroke, who were randomly assigned to the test (*n* = 60) or control groups (*n* = 60). Both groups were provided with a 3-week rehabilitation program for 2 h a day. While the control group received traditional physiotherapy, the test group additionally underwent biofeedback training. Examinations were performed on the first day and the final day of the 3-week intervention program. The primary measurement included assessment of hand grip strength (key, three jaw chuck, tip-to-tip) using an electronic dynamometer and a Biometrics E-link pinchmeter. Secondary outcomes included hand motor function assessment, using the Fugl–Meyer Assessment-Hand Function scale, hand motor dexterity with the Box and Blocks test, hand grip functions according to the Frenchay scale, and functional fitness with the Barthel index.

**Results:**

In the test group, significant rehabilitation effects were observed for the assessment of grip strength, finger compression strength, manual hand dexterity, grip function and activities of daily living (*p* < 0.001; *p* = 0.001), while in the control group results were improved for grip strength and finger compression strength (key and three-jaw chuck) of the right hand (*p* = 0.012; *p* = 0.017; *p* = 0.001) and manual dexterity (*p* < 0.001), motor abilities and activities of daily living (*p* < 0.001).

**Conclusion:**

The study showed positive effects of hand function rehabilitation in chronic stroke patients in both groups. However, in the test group, which additionally underwent training that stimulated the central nervous system using the biofeedback method with the Biometrics device, better hand and finger grip function as well as hand motor and manual function were noted.

**Clinical trial registration:**

https://clinicaltrials.gov/, Identifier NCT05486052.

## 1 Introduction

Stroke is one of the leading causes of disability worldwide. People who survive a stroke show up to 80% of motor disorders in the upper limb and hand (1, 2).

Impairment of hand function makes it difficult to perform activities of daily living. The upper limb is one of the most utilized parts of the body, therefore regaining its function should be treated as a priority in brain injury. However, its improvement is considered one of the most difficult in the rehabilitation process, because complications after a stroke include impaired sensation, limitation of motor functions, dexterity, coordination, abnormal muscle strength and tension, which disrupt the ability of such people to function in society (3–5). New methods and various techniques of working with stroke patients are mainly aimed at improving this ability.

Brain plasticity is the ability to permanently transform the brain at the level of structures and functions based on information supplied to it, which allows us to learn, remember, and undergo developmental and compensatory changes. Research confirms that neurogenesis is constantly occurring in the brain, thanks to which a stroke can be followed by a brain healing effect, so-called neural network reprogramming, potentially leading to faster recovery (6–8). The biofeedback phenomenon is related to processes that take place throughout the body, and so it can be used to stimulate a patient’s nervous system during treatment after stroke. The use of a biofeedback mechanism allows feedback to be provided to the patient, teaching them how to perform movements correctly, which is an important element of therapy when motor deficits occur (8, 9). Research shows that the use of biofeedback in robotic devices facilitates the phenomenon of brain neuroplasticity through multiple repetitions of a given movement, affecting the sensorimotor cortex (10).

Among the various techniques for using surrogate feedback, visual and auditory afferentation are most often used. This type of rehabilitation facilitates intensive training, which enables adaptation to new conditions. Research shows that working with such equipment not only improves motivation to exercise but also supports regeneration. Supplementing rehabilitation with modern devices supports the process of helping patients after stroke to regain functional fitness. In the case of functional disorders of the hand or the entire upper limb following a stroke, biofeedback methods are applied using devices such as: Biometrics (11), Armeo (12, 13), Luna (14, 15), Pablo (9) Gloreha glove (16), HandTutor (17), and Amadeo (18). Many researchers use robotic biofeedback devices to treat stroke patients, but most focus on the entire upper limb (19, 20).

The Biometrics device is a diagnostic and measurement tool that uses the biofeedback method as the basis of hand and finger grip strength exercises that make it possible to perform training to restore hand dexterity and the function of the entire upper limb (12, 21). Biofeedback allows the patient to visualize movements they are performing, which has a positive effect on engagement and increases the range of movement and muscle strength. The Biometrics device has been used many times by researchers for rehabilitation of patients with various disorders (22, 23).

A literature review showed that the Biometrics LTD device has been used in the treatment of patients with rheumatoid arthritis (24), cerebral palsy (25), and studies have been conducted using the device to improve the performance of prosthetic hands (26) as well as with spinal cord injury (27). However, no studies were found on the rehabilitation of chronic stroke patients. Although chronic stroke patients typically exhibit limited potential for recovery due to plateaued neuroplastic processes, biofeedback-based therapy may still activate residual neuroplasticity through targeted, repetitive, and feedback-driven training. Therefore, the aim of our study was to assess the effects of rehabilitation using the Biometrics device on the re-education of hand function in chronic stroke patients.

## 2 Materials and methods

### 2.1 Study design

The research was conducted as a two-group randomized controlled trial.

The research received a positive opinion from the Local Bioethics Commission of the University (No. 2022/085). The study was registered in the clinical trials register at the site ClinicalTrials.gov (registration number NCT05486052). Registration date (18.07.2022). The data were collected from 1 August 2022 to 28 February 2023.

### 2.2 Setting

The study was conducted among chronic stroke patients in the Spa and Rehabilitation Hospital.

### 2.3 Sample size calculation

The required sample was taken *a priori* based on the minimal clinically important difference (MCID) for the FMA-UE scale, 4.25 points (28). Using the G*Power program (version 3.1.9.4; F. Faul, University of Cologne, Germany), with a statistical power of 90% (1-*β*) and a significance level of *α* = 0.05, a minimum required sample size was calculated as 38 participants in each group (76 in total). However, due to the specific characteristics of those examined, more conservative assumptions regarding oversampling (approximately 25–30%) were deliberately made. 120 patients were enrolled in the study. After applying a 0% elimination estimate, the final sample size, distributed across units, ultimately yielded very high statistical power.

### 2.4 Study population

Chronic stroke patients were randomly assigned to two groups (test and control). Randomization was performed by the double-blind method, in which both participants and outcome assessors were blinded to group allocation. Due to the nature of the intervention, the therapists administering the treatment were not blinded. Randomization was performed using a computer-generated random number sequence created in Microsoft Excel (Microsoft Corp., Redmond, WA, USA). To ensure concealment of allocation, sealed, opaque envelopes were prepared by an investigator not involved in participant recruitment or outcome. The inclusion criteria were: first ischemic stroke, medical examination confirming participation in exercises, basic gripping ability for the upper limbs and hand, modified Ashworth scale not higher than 3, Brunnström scale 4–5, Rankin scale 3, time since stroke greater than 6 months, written informed consent to participate in the study. The exclusion criteria were: non-ischemic stroke, lack of informed consent from the patient to participate in the study, mechanical, thermal injuries and comorbidities that may impair hand-grip function, unstable health state.

### 2.5 Interventions

**Figure 1:**
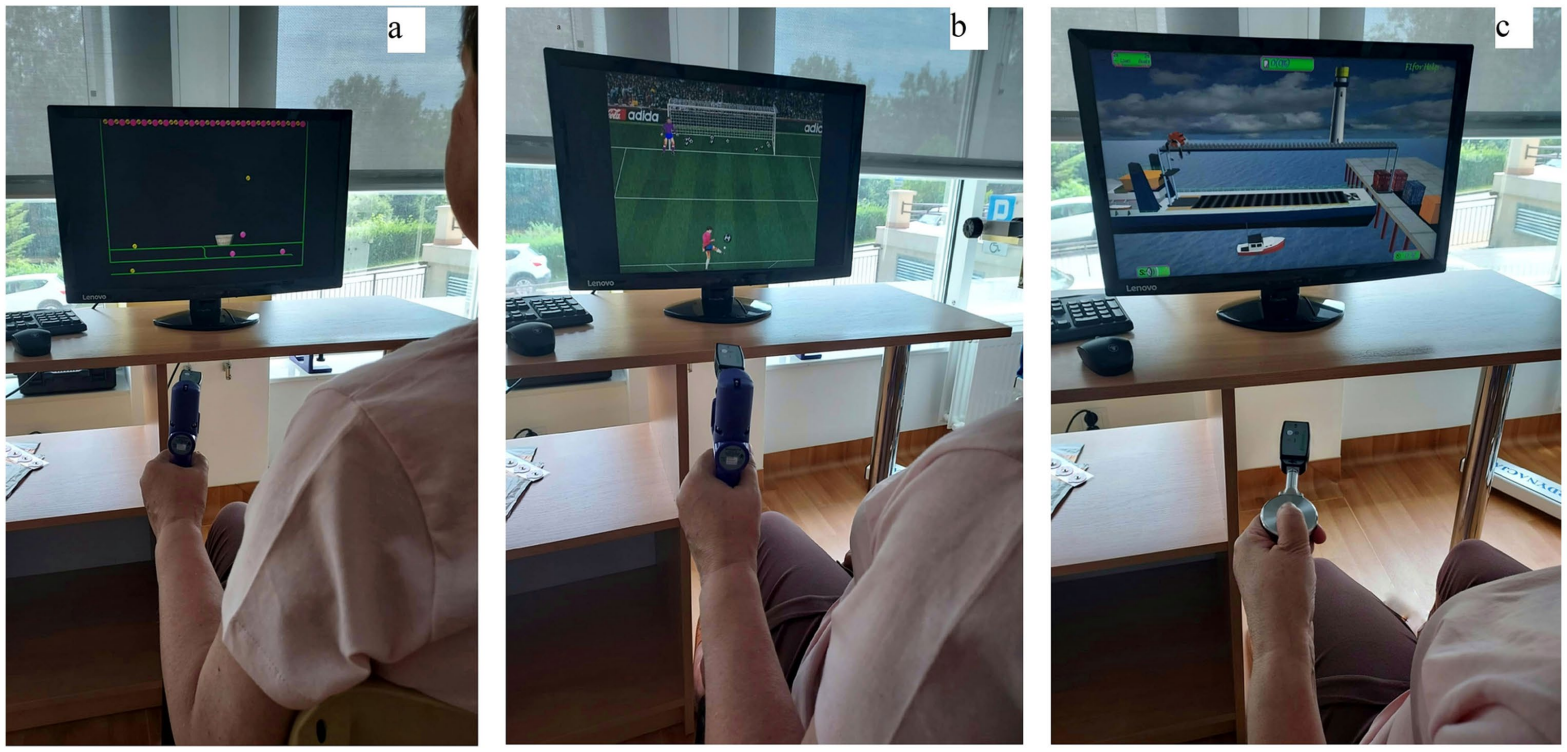
Biofeedback exercises: **(a)** catching the correct colors of balls for a basket, **(b)** shooting balls into a goal, **(c)** laying colored blocks.

The rehabilitation program lasted 3 weeks (from Monday to Friday) and took 2 h a day for both groups. All patients participated in individual and group exercises, massages, physical treatments and treatments using natural resources. In addition, patients in the test group participated in 30 min per day of exercises using a biofeedback method, which were performed on the Biometrics dynamometer device and aimed at improving hand motor function (29). These activities were conducted within the existing 2-h sessions, rather than as extra therapy time. The training took place on the basis of tasks stimulating the central nervous system with the help of visual and acoustic biofeedback. These tasks consisted of catching the correct colors of balls for a basket (a), shooting balls into a goal (b), laying colored blocks (c) (Figure 1).

### 2.6 Outcome measures

The first examination was performed on the first day of rehabilitation, and the second on the last day of the 3-week intervention program (at discharge).

The primary measurement of hand grip and finger strength (key, three jaw chuck, tip-to-tip) was based on an objective method, using an electronic dynamometer and a Biometrics E-link pinchmeter. The former registers forces from less than 0.1 kg/lb. to 90 kg (200 lb) while the latter records finger pinch strength up to 22 kg (50 lb) (29, 30).

All measurements were taken according to the American Society of Hand Therapists (ASHT) guidelines and the Biometrics E-link device reliability assessment methodology for assessing hand grip and finger pinch strength in healthy individuals (29, 31).

Secondary results included an assessment of hand motor function, performed using the Fugl–Meyer Assessment-Hand Function (FMA-Hand) scale (32) to assess precise movements and grips: motor control of finger flexion and extension, thumb adduction, finger resistance, cylindrical and spherical grip. The patient received 2 points for making a full movement, 1 point for a partial movement, 0 points for no movement. The Fugl–Meyer Motor Assessment Scale for Upper Extremity (FMA-UE) consists of 7 items (FMA-UE headings 24 to 30) giving a maximum possible 14 points (5, 33–35). Hand motor skills were assessed using the Box and Blocks (BBT) test. The patient was asked to move as many blocks (2.5 cm) as possible in a wooden box (53.7 cm x 25.4 cm x 8.5 cm) divided by a partition into two parts within 60 s. The higher the number of blocks moved, the better the patient’s manual hand dexterity (36–38). Hand grip was assessed according to the Frenchay scale, which consists of 7 tasks, for which the patient receives 1 point when performing them correctly, and 0 points for failing to perform them, giving a maximum possible 7 points. The higher the patient’s score, the better their hand dexterity (39, 40). The patients’ functional status was assessed using the Barthel Index (BI), which consists of 10 items assessing activities of daily living. The results of all items are added together to determine the patient’s condition: 86–100 pts. – patient’s condition “light”; 21–85 points – patient’s condition “moderately severe”; 0–20 points – patient’s condition “very severe” (41–43).

### 2.7 Data analysis

Statistical analysis of the collected material was performed in the Statistica 13.3 package. The database and the graphical elaboration of the results were prepared in Microsoft Excel and Microsoft Word.

Descriptive statistics were calculated: number, mean value, median, minimum and maximum values, upper and lower quartile and standard deviation. For the assessment of statistical differences between the test and control groups in the first and second examinations, and for the assessment of the effects of rehabilitation, the student-*t* test for independent samples was used, or due to non-compliance with the assumptions of parametric tests (lack of compliance of the variable distribution with the normal distribution verified by the Shapiro–Wilk test or a dependent variable of an ordinal character), the non-parametric Mann Whitney U test was used. The Wilcoxon non-parametric test was used to assess the effects of rehabilitation between the first and second examinations in the test and control groups because of the lack of compliance of the distribution of differences with the normal distribution.

The level of statistical significance was *p* < 0.05.

## 3 Results

**Figure 2:**
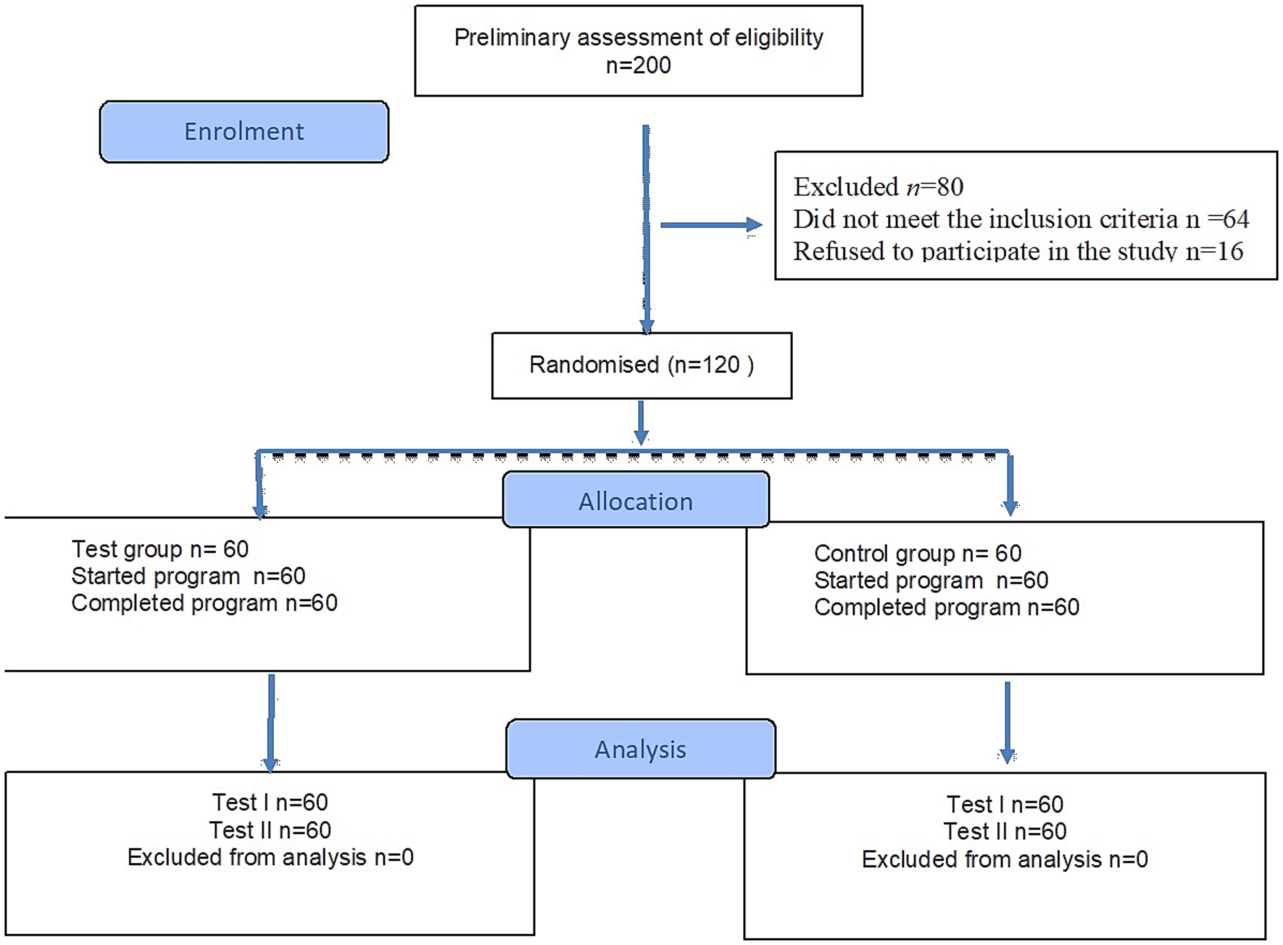
Flow diagram.

Among 200 patients admitted to the Spa and Rehabilitation Hospital, preliminary qualification was performed based on the criteria of inclusion and exclusion for the study. 64 people did not meet the criteria, and 16 people did not agree to participate in the study. The study included 120 stroke patients (n = 60 test group, n = 60 control group) who completed the 3-week rehabilitation program (Figure 2).

In both groups, a slight and insignificant majority consisted of men (66.7 and 65.0%) and people with left-sided paresis (58.3% each). The mean age in the test group was x̄ = 62.7, and in the control group x̄ = 63.6. The mean time since stroke in the test group was x̄ = 43.4 months and in the control group x̄ = 43.5 (Table 1).

**Table 1:** Differences in age, body weight [kg], body height [cm], BMI and time since stroke in the test group and control group.

Characteristics	Test group (*n* = 60)	Control group (*n* = 60)	*p*-value
Age (years)	62.7 (7.80)	63.6 (6.04)	0.551
	63.5 (55.5–69.5)	64.5 (59.0–68.0)	
Body weight (kg)	76.4 (11.09)	77.3 (12.01)	0.633
	77.8 (69.1–81.2)	78.8 (70.2–83.2)	
Body height (cm)	170.3 (7.21)	169.1 (6.98)	0.364
	170.0 (165.0–176.0)	169.0 (164.0–173.0)	
BMI (kg/m^2^)	26.3 (3.28)	27.0 (3.43)	0.300*
	26.8 (24.8–28.0)	26.6 (24.9–28.7)	
Time since stroke (months)	43.4 (45.63)	43.5 (43.27)	0.904
	24.0 (10.5–60.5)	23.5 (10.0–77.0)	

Data are presented in two rows: the first row shows Mean (Standard Deviation), and the second row shows Median (Lower Quartile – Upper Quartile).

*p*-values were calculated using the independent *t*-test* or Mann–Whitney U test to compare the test and control groups at baseline.

The analysis of the results from the first (baseline) study did not show statistically significant differences between the test and control groups in any of the evaluated demographic and clinical variables (*p* > 0.05 for all comparisons). The groups were comparable in terms of age, body weight, BMI, and time since stroke. Similarly, no significant between-group differences were found in baseline measurements of grip and finger pinch strength, manual dexterity, or functional assessments. This confirms that the randomization process successfully created two homogeneous groups before the intervention began.

Using a dynamometer and a pinchmeter, respectively, hand grip strength and finger compression strength (key) in the test and control groups were examined in the first and second examinations. In both groups, the effects of rehabilitation on the right hand were shown to be effective (*p* < 0.001; *p* = 0.012). Left hand scores were significantly improved only in patients participating in rehabilitation supplemented with biofeedback therapy (*p* < 0.001), which also translated into better rehabilitation outcomes (*p* < 0.001) in this group (Table 2).

**Table 2:** Comparison of hand grip and key pinch strength outcomes.

Variable	Group	Baseline	Post-intervention	*p*-value^a^	*p*-value^b^
Hand grip strength Right hand (kg)	Test (*n* = 60)	23.6 (12.76)	28.9 (15.45)	<0.001	0.246
		21.3 (17.6–28.0)	26.7 (20.0–34.5)		
	Control (*n* = 60)	20.3 (12.55)	23.6 (17.39)	0.012	
		18.4 (12.5–24.0)	19.4 (10.0–32.3)		
Hand grip strength - Left hand (kg)	Test (*n* = 60)	20.2 (17.55)	25.3 (19.41)	<0.001	0.001
		15.1 (8.8–26.7)	22.7 (13.8–28.7)		
	Control (*n* = 60)	20.8 (10.59)	21.8 (12.21)	0.213	
		20.0 (13.0–28.8)	18.6 (12.0–29.6)		
Key pinch - Right hand (kg)	Test (*n* = 60)	6.4 (4.43)	8.3 (6.08)	<0.001	0.159
		5.9 (4.1–7.8)	7.2 (5.5–9.0)		
	Control (*n* = 60)	5.7 (3.91)	6.4 (4.06)	0.017	
		5.0 (3.3–6.9)	5.0 (3.9–8.3)		
Key pinch - Left hand (kg)	Test (*n* = 60)	5.3 (4.42)	6.8 (5.12)	<0.001	0.008
		3.9 (2.4–6.7)	5.9 (3.3–8.3)		
	Control (*n* = 60)	5.9 (3.44)	6.2 (3.75)	0.433	
		5.4 (3.3–7.0)	5.1 (3.9–8.1)		

Data are presented in two rows for each group: the first row shows Mean (Standard Deviation), and the second row shows Median (Lower Quartile – Upper Quartile).

*p*-value^a^ for within-group changes was calculated using the Wilcoxon signed-rank test (Baseline vs. Post-intervention).

*p*-value^b^ for the comparison of changes between groups was calculated using the Mann–Whitney U test.

There were no inter-group differences (*p* > 0.05) for the finger compression strength (three-jaw chuck) assessment. After the therapy, the results for the right hand were significantly improved in both groups (*p* < 0.001; *p* = 0.001), while for the left hand only in people undergoing rehabilitation supplemented with biofeedback (*p* = 0.001). A similar situation is presented in assessment of the results of the finger compression strength (tip-tip) in the right and left hands. Only in the test group were significant effects of rehabilitation reported (*p* < 0.001). After the second measurement, people in the test group were characterized by higher finger compression strength in the right hand than people in the control group. In the left hand, the effects of therapy were more beneficial in the test group than in the control group (*p* = 0.017) (Table 3).

**Table 3:** Comparison of finger pinch strength outcomes.

Variable	Group	Baseline	Post-intervention	*p*-value^a^	*p*-value^b^
Three-jaw chuck - Right hand (kg)	Test	6.4 (4.58)	7.8 (5.28)	<0.001	0.808
		5.3 (4.0–7.8)	7.0 (4.3–9.3)		
	Control	5.3 (3.76)	6.5 (4.91)	0.001	
		4.6 (2.4–6.7)	4.7 (3.5–7.6)		
Three-jaw chuck - Left hand (kg)	Test	5.4 (4.41)	6.5 (4.50)	0.001	0.237
		4.0 (2.2–7.6)	5.0 (3.8–7.8)		
	Control	5.2 (3.35)	5.8 (3.64)	0.129	
		4.5 (3.1–6.9)	4.7 (3.5–8.3)		
Tip to tip pinch - Right hand (kg)	Test	5.8 (4.31)	6.5 (4.09)	<0.001	0.199
		4.8 (3.7–6.6)	5.8 (4.4–7.2)		
	Control	4.5 (2.57)	5.0 (2.98)	0.054	
		4.2 (2.8–5.3)	4.2 (2.8–6.0)		
Tip to tip pinch - Left hand (kg)	Test	4.2 (3.00)	5.4 (3.62)	<0.001	0.017
		3.4 (2.0–5.2)	4.4 (3.0–6.9)		
	Control	4.3 (2.83)	4.6 (2.51)	0.138	
		3.7 (2.2–6.1)	3.9 (2.8–6.1)		

Data are presented in two rows for each group: the first row shows Mean (Standard Deviation), and the second row shows Median (Lower Quartile – Upper Quartile).

*p*-value^a^ for within-group changes was calculated using the Wilcoxon signed-rank test (Baseline vs. Post-intervention).

*p*-value^b^ for the comparison of changes between groups was calculated using the Mann–Whitney U test.

In terms of manual hand dexterity, it was noted that both conventional and biofeedback rehabilitation brought the assumed benefits (*p* < 0.001). The effects of the therapy on the non-dominant hand were significantly better in the test group (*p* = 0.028). Hand grip function and hand motor abilities were significantly improved in both groups after measurement II (*p* < 0.001). Significantly better results of therapy were reported in people using rehabilitation supplemented with biofeedback. The final tool used was the Barthel scale, which assessed activities of daily living. There were no inter-group differences in either the first or second examinations, or in the effect assessment, but it was noted that both groups obtained more favorable results after rehabilitation than before the therapy (*p* < 0.001) (Table 4).

**Table 4:** Comparison of functional and dexterity outcomes.

Variable	Group	Baseline	Post-intervention	*p*-value^a^	*p*-value^b^
Box & Blocks - Dominant hand (blocks)	Test	30.9 (13.01)	35.1 (13.76)	<0.001	0.267
		32.0 (24.0–40.0)	37.5 (25.5–44.5)		
	Control	28.6 (11.06)	32.4 (13.20)	<0.001	
		29.0 (21.0–34.0)	31.5 (23.5–39.5)		
Box & Blocks - Non-dominant hand (blocks)	Test	26.5 (11.95)	31.3 (12.27)	<0.001	0.028
		25.5 (17.0–33.0)	30.0 (23.0–37.0)		
	Control	27.6 (10.71)	30.1 (10.48)	<0.001	
		26.0 (20.0–34.0)	29.5 (23.0–36.0)		
Frenchay scale (points)	Test	4.1 (0.91)	5.5 (0.69)	<0.001	<0.001
		4.5 (3.3–5.0)	5.5 (5.0–6.0)		
	Control	4.4 (0.88)	5.2 (0.89)	<0.001	
		4.5 (4.0–5.0)	5.5 (4.5–6.0)		
Fugl-Meyer scale (points)	Test	8.8 (1.28)	10.3 (1.39)	<0.001	<0.001
		9.0 (8.0–9.0)	11.0 (9.0–11.0)		
	Control	9.1 (1.62)	9.6 (1.63)	<0.001	
		9.0 (9.0–10.0)	10.0 (9.0–11.0)		
Barthel Index (points)	Test	80.6 (8.34)	85.5 (7.46)	<0.001	0.053
		85.0 (75.0–85.0)	87.5 (80.0–90.0)		
	Control	79.1 (8.26)	82.4 (8.71)	<0.001	
		80.0 (75.0–85.0)	85.0 (75.0–90.0)		

Data are presented in two rows for each group: the first row shows Mean (Standard Deviation), and the second row shows Median (Lower Quartile – Upper Quartile).

*p*-value^a^ for within-group changes was calculated using the Wilcoxon signed-rank test (Baseline vs. Post-intervention).

*p*-value^b^ for the comparison of changes between groups was calculated using the Mann–Whitney U test.

## 4 Discussion

The main aim of the study was to check the effects of rehabilitation of chronic stroke patients in terms of changes in hand motor function and self-reliance, and then to determine the differences in these effects depending on the method used, i.e., biofeedback method and conventional method. The study showed that patients improved manual dexterity in both the test and control groups, but better effects of therapy were noted for the group of patients using rehabilitation combined with biofeedback.

A similar study was conducted by Dziemian et al., using biofeedback exercises during hand rehabilitation. As in our own study, a statistically significant improvement in hand function was shown on the Fugl–Meyer scale and manual hand dexterity in the Box and Blocks test after biofeedback therapy in the test group. The authors draw conclusions on the benefits of implementing biofeedback therapy to improve impaired upper limb function. However, it is worth adding that the study included only 10 patients after stroke, including 8 after ischemic stroke and 2 after haemorrhagic stroke, so the group was heterogeneous and small (44). In our own study, we examined a relatively large and homogeneous group of 120 patients in the chronic phase after a single ischemic stroke, all at a similar motor recovery level. This sample size strengthens the reliability of our findings regarding the positive effects of biofeedback in post-stroke rehabilitation.

In our own study, in measurements using a dynamometer and pinchmeter, patients who used biofeedback equipment also performed better. The analysis of the finger compression strength measurements was carried out in three positions – key, tip-tip, three-jaw chuck. An important element of the study was to record improvements in finger compression strength (in the key and three-jaw chuck positions) for patients who received biofeedback-enhanced therapy. Similar effects were noted for the tip-tip compression strength. Bayidir et al. also assessed grip and finger pinch strength (tip-tip) in a randomized-controlled study of patients following stroke, and their results confirmed better effects for a group of patients who received biofeedback-enriched therapy (17). Hsu et al. studied the effects of robot-assisted training, in combination with conventional rehabilitation, on hand function chronic stroke patients. Their observations showed an improvement in hand function. However, the test group was small, at just 12 people, so the authors recommend further research to confirm the validity of their reports (45).

The above results of both our own and other authors’ research indicate that the use of biofeedback methods together with traditional rehabilitation gives good therapeutic effects in terms of manual function and hand grip strength in chronic stroke patients. It is worth adding, however, that there are conflicting reports in the literature regarding rehabilitation using biofeedback methods. It should be noted that although significant differences were obtained, we did not obtain clinically important differences (MCID). The MCID for the FMA = grasping ability 4.25, releasing ability 5.25 (28), and our patients improved by 1.28–1.63 points. The late post-stroke period in which our subjects were present may have influenced this result. However, we noted the need for continued exercise and ongoing hand rehabilitation in our patients. When analyzing the literature on this topic, numerous studies indicate positive effects from using robotic equipment with biofeedback for improving upper limbs, including the hand, after a stroke (19, 46–49). However, while some studies unambiguously confirm the benefits of robotic therapy, others find no significant differences in fitness improvement between classical rehabilitation and biofeedback methods (50). Therefore, there was a need and rationale to conduct this study in a homogeneous group of stroke patients in order to assess the effects of hand rehabilitation using biofeedback methods. The obtained results allowed us to confirm the hypothesis of higher effectiveness of biofeedback methods compared to the conventional method in hand rehabilitation of chronic stroke patients. The practical application of these studies will enable the development of rehabilitation programs for chronic stroke patients, in whom the adaptive use of a fixed movement pattern may have occurred. The observed advantage of biofeedback-based therapy can be explained by its underlying neurophysiological mechanisms. Firstly, sensory feedback – visual, auditory or proprioceptive information – provided during exercise, improves sensorimotor integration and enables error correction, which promotes the phenomenon of neuroplasticity – the strengthening of synaptic connections through the interaction of sensory and motor cortices. A review of biofeedback studies for neuromotor rehabilitation by Huang et al. showed that biofeedback can increase plasticity by engaging additional sensory stimuli (51). Second, biofeedback requires intense attentional engagement, which modulates cortical excitability and facilitates learning-dependent changes in the motor cortex. A review by Proulx et al. on somatosensory, visual, and auditory feedback and their interactions applied to upper limb neurorehabilitation technology showed that the response of the somatosensory cortex is crucial for improving motor skills after stroke (52). Third, task repetition and specificity are key indicators of neuroplasticity for motor learning. Research indicates that repetition of intentional movements leads to long-term potentiation, reorganization of cortical maps and unblocking of synaptic latencies, even in chronic stages after stroke (53–55).

Importantly, our study found significant functional improvements in people in chronic stages after stroke, a population often thought to have reached a plateau in functional recovery. However, abundant scientific evidence from biofeedback shows that plasticity still exists when a rich sensory environment and task-specific training are provided (56–60). The mechanism of action includes cortical remapping, thanks to activity dependent on the type of training and repetition, additionally sensory stimulation and exercise increase connectivity in the somatomotor network, (53). Biofeedback therapy, by combining sensory stimuli, cognitive engagement, and intense motor repetition, likely reactivates dormant neural pathways and promotes reorganization in cortical areas. This provides a physiological rationale for the observed improvement and supports the inclusion of biofeedback even in rehabilitation protocols for the late stage after stroke.

### 4.1 Limitations

One of the limitations is the age of the patients, as it was conducted in people over 50 years of age. Scientific reports show that stroke occurs more often in people over that age, but since the incidence of stroke is becoming more frequent among younger people, they should also be included in further studies. Another limitation is the time since stroke. The studies were conducted among chronic stroke patients, a group of patients that is not often analyzed in terms of improving their fixed hand patterns. Further studies should also include people in the early period and also analyze their hand function during this time. Although both groups received the same total duration of therapy (2 h per day over 3 weeks), the integration of biofeedback within the intervention sessions may have introduced differences in cognitive engagement or patient motivation. This potential influence, while not related to therapy time per se, could be considered a confounding factor and should be taken into account when interpreting the superiority of biofeedback-based rehabilitation. Further studies are warranted to isolate the specific contribution of biofeedback mechanisms.

## 5 Conclusion

The study showed positive effects of hand function rehabilitation in chronic stroke patients in both groups. However, in the test group, which additionally underwent training that stimulated the central nervous system using the biofeedback method with the Biometrics device, better hand and finger grip function as well as hand motor and manual function were noted. Therefore, it can be concluded that exercises using the Biometrics device have clinical application in the re-education of hand function.

## Data Availability

The original contributions presented in the study are included in the article/supplementary material, further inquiries can be directed to the corresponding author.
